# Supplementary material for: Health Benefits of Whey or Colostrum Supplementation in Adults ≥35 Years; a Systematic Review
Source: Nutrients. 2020 Jan 22;12(2):299. doi: 10.3390/nu12020299 (PMC7070284; doi:10.3390/nu12020299)
Supplement: Supplementary file 1 [file nutrients-12-00299-s001.pdf]

Supplementary Data

Supplemental Table 1. Example search strategy

Ovid Medline (searched 5/9/2018) – no limitations or restrictions

Adult OR adults	Colostrum OR<br>protein OR<br>proteins OR<br>whey OR<br>casein	Dairy<br>OR milk

Adult OR adults AND Colostrum OR protein OR proteins OR whey OR casein AND Dairy OR milk

Supplemental Table 2. Risk of bias summary of included papers using the Cochrane Risk of Bias tool V2.0[1]

	Domain 1	Domain 2	Domain 3	Domain 4	Domain 5	Risk of bias judgement
Aoe 2005 [38]	+	+	?	+	?	Some concerns
Arciero 2014 [31]	?	−	−	+	+	High
Baer 2011 [32]	?	?	−	+	?	High
Bemben 2010 [30]	?	?	?	+	?	High
Chale 2013 [41]	+	+	+	+	+	Low
Duff 2014 [40]	+	+	+	+	?	Some concerns
Devries 2018 [39]	+	?	+	+	+	Some concerns
Eliot 2008 [29]	?	?	+	+	?	Some concerns
Farnfield 2012 [33]	?	?	?	+	?	High
Francis 2017 [34]	?	?	?	+	+	High
Frestedt 2008 [35]	?	?	−	+	−	High
Hector 2015 [36]	?	?	?	+	+	High
Kerstetter 2015 [42]	+	+	+	+	+	Low
Norton 2016 [37]	?	?	−	+	+	High
Pal 2010a [27]	?	+	?	+	+	Some concerns
Pal 2010b [28]	?	?	?	+	+	High

** Any paper with 3 or more 'some concerns' was determined to be high risk of bias **

KEY:

+ Low risk of bias

? Some concerns

− High risk of bias

[1] Sterne J, Savovic J, Page M. RoB 2: a revised tool for assessing risk of bias in randomised trials. BMJ.

Domain 1 – Risk of bias arising from the randomisation process
Domain 2 – Risk of bias due to deviation from the intended intervention (effect of assignment to intervention)
Domain 3 – Missing outcome data
Domain 4 – Risk of bias in measurement of the outcome
Domain 5 – Risk of bias in selection of the reported result
